# Short-term vasoactive agent treatment driven by physicians’ preference in acute esophageal variceal bleeding in a tertiary center

**DOI:** 10.7717/peerj.7913

**Published:** 2019-11-06

**Authors:** Yoen Young Chuah, Ping-I Hsu, Wei-Lun Tsai, Hsien-Chung Yu, Feng-Woei Tsay, Wen-Chi Chen, Kung Hung Lin, Yeong Yeh Lee, Huay-Min Wang

**Affiliations:** 1 Division of Gastroenterology and Hepatology, Department of Medicine, Kaohsiung Veterans General Hospital, Kaohsiung, Taiwan; School of Medicine, National Yang-Ming University, Taipei, Taiwan; 2 Division of Gastroenterology and Hepatology, Department of Medicine, Ping Tung Christian Hospital, Ping Tung, Taiwan; Department of Nursing, Meiho University, Taiwan; 3 School of Medical Sciences, Universiti Sains Malaysia, Kota Bharu, Malaysia; 4 Gut Research Group, Faculty of Medicine, The National University of Malaysia, Kuala Lumpur, Malaysia

**Keywords:** Terlipressin, Somatostatin, Esophageal variceal bleeding, Physician preference

## Abstract

**Background:**

Vasoactive drugs are frequently used in combination with endoscopic variceal ligation (EVL) in treatment of acute esophageal variceal bleeding (EVB). The aim of study was to assess physicians’ preference of vasoactive agents in acute EVB, their reasons of preference and efficacy and safety of these short course regimens.

**Methods:**

Cirrhotic patients with suspected EVB were screened (*n* = 352). Eligible patients were assigned based on the physician’s preference to either somatostatin (group S) or terlipressin (group T) followed by EVL. In group S, intravenous bolus (250 µg) of somatostatin followed by 250 µg/hour was continued for three days. In group T, 2 mg bolus injection of terlipressin was followed by 1 mg infusion every 6 h for three days.

**Results:**

A total of 150 patients were enrolled; 41 in group S and 109 in group T. Reasons for physician preference was convenience in administration (77.1%) for group T and good safety profile (73.2%) for group S. Very early rebleeding within 49–120 h occurred in one patient in groups S and T (*p* = 0.469). Four patients in group S and 14 patients in group T have variceal rebleeding episodes within 6–42 d (*p* = 0.781). Overall treatment-related adverse effects were compatible in groups S and T (*p* = 0.878), but the total cost of terlipressin and somatostatin differed i.e., USD 621.32 and USD 496.43 respectively.

**Conclusions:**

Terlipressin is the preferred vasoactive agent by physicians in our institution for acute EVB. Convenience in administration and safety profile are main considerations of physicians. Safety and hemostatic effects did not differ significantly between short-course somatostatin or terlipressin, although terlipressin is more expensive.

## Background

Rupture of esophageal varices that results in variceal hemorrhage is a major complication associated with high mortality rate (Garceau & Chalmers, 1963; Graham & Smith, 1981). Despite initial control of bleeding, early rebleeding rate may be as high as 30–50% among the survived patients and the mortality rate can be up to 40% (Garcia-Tsao et al., 2007). Therefore, it is recommended that treatment goals should aim for bleeding arrest and prevention of early rebleeding (D’Amico, Pagliaro & Bosch, 1995).

Vasoactive drugs may reduce portal hypertension which lead to a reduction in variceal pressure to achieve better control of hemorrhage (Garcia-Pagan et al., 1999; Villanueva et al., 2001). In general, vasoactive agents including somatostatin and terlipressin were highly effective in control of variceal bleeding if compared to the placebo (D’Amico, Pagliaro & Bosch, 1999). On the other hand, the efficacy of somatostatin for early rebleeding control has been reported to be similar to terlipressin (D’Amico, Pagliaro & Bosch, 1999; Burroughs et al., 1990; Cales et al., 2001; Chen et al., 2006; Feu et al., 1996; Levacher et al., 1995). In suspected acute variceal bleeding (EVB), vasoactive drugs should be started as soon as possible and are generally prescribed for 5 days to prevent against very early rebleeding (Lo, 2010; De Franchis, 2005). Endoscopic variceal ligation (EVL) is recommended in patients with EVB and is best used in combination with vasoactive drugs according to the Baveno IV consensus. Short-course administration (2-day or 3-day) of vasoactive drugs have been shown to be as effective as a 5-day course for the control of acute EVB when used as an adjuvant therapy to EVL (Chitapanarux et al., 2015; Rengasamy et al., 2015).

The management of acute EVB in real-world clinical practice may be different from clinical trials or from available guidelines. In Taiwan, the National Health Insurance (NHI) program uses a single-payer system and covers 99.9% of the nation’s population and therefore provides their physicians a high degree of freedom in their choice of medications (Li et al., 2015). The Taiwan NHI program approves short-course (3 days) administration of any two vasoactive drugs i.e., somatostatin or terlipressin in treating acute EBV. The aim of study was to assess the physicians’ preference of vasoactive agents, their reasons of preference and efficacy and safety of short-course regimen of these agents in the real-world clinical practice.

## Methods

### Study patients

From April 27, 2010 through April 26, 2015, cirrhotic patients who were admitted to Kaohsiung Veterans General Hospital (tertiary referral center) and with acute upper gastrointestinal bleeding were screened (*n* = 352). Eligible patients with acute EVB were non-randomly assigned to receive a 3-day course of either somatostatin (group S) or terlipressin (group T) based on preference of treating physician followed by EVL within 24 h.

Inclusion criteria were: (i) acute hemorrhage from esophageal varice(s) defined as oozing or spurting directly observed endoscopically, or when red color signs and/or white nipple sign were seen on esophageal varices without any other potential sources of bleeding; (ii) portal hypertension attributed to liver cirrhosis of any cause; (iii) adults with age between 20 and 80 years old.

Patients were excluded if they met the following criteria: (i) hepatocellular carcinoma (HCC) of Barcelona Clinic Liver Cancer (BCLC) >C (ii) severe illness such as chronic obstructive pulmonary disease (COPD), septic shock, cerebral vascular event, acute coronary syndrome and uremia; (ii) with current gastric variceal bleeding; (iii) previous endoscopic injection sclerotherapy (EIS), EVL within 6 month prior to current bleeding episode; (iv) previous shunt surgery or transjugular intrahepatic porto-systemic stent shunt (TIPS) procedure; (v) allergic to and/or with contraindications to vasoactive agents; (vi) pregnancy. (vii) participation of other trials.

Adverse events were further classified into vasoactive agent-related and EVL related. The former consisted of chest tightness/pain, hypertension (defined as SBP > 140 mmHg), abdominal pain, arrhythmia, hyponatremia (Na < 125 mmol/L), lymphangitis, renal failure (Cr > 2 mg/dL), hyperglycemia (Glu > 300 mg/dL) and flush. The later included chest tightness/pain, retrosternal pain, dysphagia/odynophagia, post-EVL ulcer and esophageal stricture.

Informed consent was sought from each patient in written consent form before administration of vasoactive agents. This study conformed to the Declaration of Helsinki of the World Medical Association (2008) and it was approved by the Institutional Review Board at Kaohsiung Veterans General hospital, Taiwan, Republic of China (IRB No. VGHKS99-CT4-20). The clinical trial identification number is NCT02757703 (ClinicalTrials.gov Identifier). However, the protocol was modified after the initial submission in clinical trial registration with the definition of hypertension changed to SBP>140 mmHg.

### Study design

In this single-center prospective cohort study, eligible patients were non-randomly assigned into two groups (group T: terlipressin and group S: somatostatin) based on the clinical choice of participating physicians (*n* = 32). Of those 32 physicians, 22 were from department of emergency and 10 were from department of gastroenterology and hepatology in our institution. They were requested to complete a questionnaire immediately after the first dose of vasoactive agent was given. This questionnaire was aimed to understand the underlying reasons for their choice of treatment (convenience in administration, good safety profile, random selection without clear reasons).

Choosing convenience in administration mean physicians were concerned about how easy the drug was to be prescribed. For instances, somatostatin was delivered intravenously in continuous running mode whereas terlipressin was prescribed in bolus form every six hours. On the other hand, physicians choosing good safety profile were more concerned about prescribing drug with less side effect.

### Study procedure

The administered vasoactive agent was either terlipressin (Glypressin, Ferring GmbH, Kiel, Germany) or somatostatin (Somatosan, BAG Health Care GmbH, Lich, Germany). Intravenous bolus of 250 µg somatostatin was given first followed by 250 µg/hour and it was continued for 3 days (group S). On the other hand, terlipressin was started at 2mg bolus injection and this was followed by 1 mg infusion every 6 h for 3 days (group T). Experienced nurse practitioners administered the drugs and subsequently they would monitor the vital signs of patients and document any side-effects. Physicians would be alerted for serious side-effects including chest discomfort and ECG changes.

After being pre-medicated with hyoscine-N-butylbromide intramuscularly (20 mg), eligible patients then underwent emergent endoscopy (GIF XQ260; Olympus Co. Ltd, Tokyo, Japan) within 12 h upon admission. Two experienced endoscopists (WCC and WMW) performed all emergent EVL with pneumoactive ligation device (Sumitomo Bakelite Co., Ltd, Tokyo, Japan) using a standard approach published previously (Wang et al., 2014). The two endoscopists had more than 10 years of experience with EVL and they were capable in handling any arising complications. These two endoscopists were not involved in the choice of vasoactive agent given to respective patients. After EVL, patients were routinely placed on liquid diet in the following 3 days and subsequently to semisolids and solids if without complications.

Pantoprazole (Pantoloc i.v., Nycomed GmbH, Singen, Germany) 40 mg was given intravenously after EVL to all study participants for 3 days, and this was followed by the oral form (Pantoloc, Takeda GmbH, Oranienburg, Germany) for 12 days in order to hasten the process of ulcer healing induced by ligation. Oral administration of non-selective *β*-blocker was usually commenced on day five. In addition, prophylactic antibiotic was initiated simultaneously with vasoactive agents and continued for 3 days. Elective repeat EVL was undertaken at the interval of three to four weeks after the index endoscopy. The period of follow-up was six weeks.

### Study end points and definitions

The study end points included (1) the control of initial bleeding (initial hemostasis), (2) very early rebleeding, (3) early rebleeding, (4) mortality at six weeks, (5) blood transfusion requirement, (6) length of hospital stays and (7) adverse events that were seen in the real-world clinical practice.

Control of initial bleeding (initial hemostasis) was defined as when treatment failure did not occur within 48 h of study recruitment. The criterion of treatment failure whichever occurred first included (1) new fresh blood vomitus ≥ 2 h after the start of vasoactive drugs or therapeutic endoscopy, (2) hemoglobin drop ≥ 3 grams per deciliter in patients not transfused and (3) death. An episode of clinically significant bleeding was defined by blood transfusion ≥ 2 units of packed red blood cells. Very early rebleeding was defined as acute variceal bleeding that occurred from 49 to 120 h after initial control of acute hemorrhage. Early rebleeding was defined as variceal hemorrhage that occurred from day 6 till day 42 after the initial bleeding arrest.

### Sample size and statistical analysis

Based on an adverse effect rate of 20% in patients treated with terlipressin and 4% in patients treated with somatostatin, a one-tailed test, allocation ratio of 2:1 to achieve power of 80% and a type I error rate of 5%, the calculated sample size was 79 cases in group T and 40 cases in group S (Rengasamy et al., 2015; Li et al., 2015; Wang et al., 2014; Seo et al., 2014). Numerical data were expressed as mean  ±  standard deviation unless stated otherwise. Univariable analysis was performed using independent *t*-test for numerical data and Chi-square or Fisher’s exact test for categorical data. *P*-value <0.05 was considered statistically significant. All analyses were conducted using SPSS 12.0.1C (SPSS Inc., Chicago, IL, USA).

## Results

**Figure 1:**
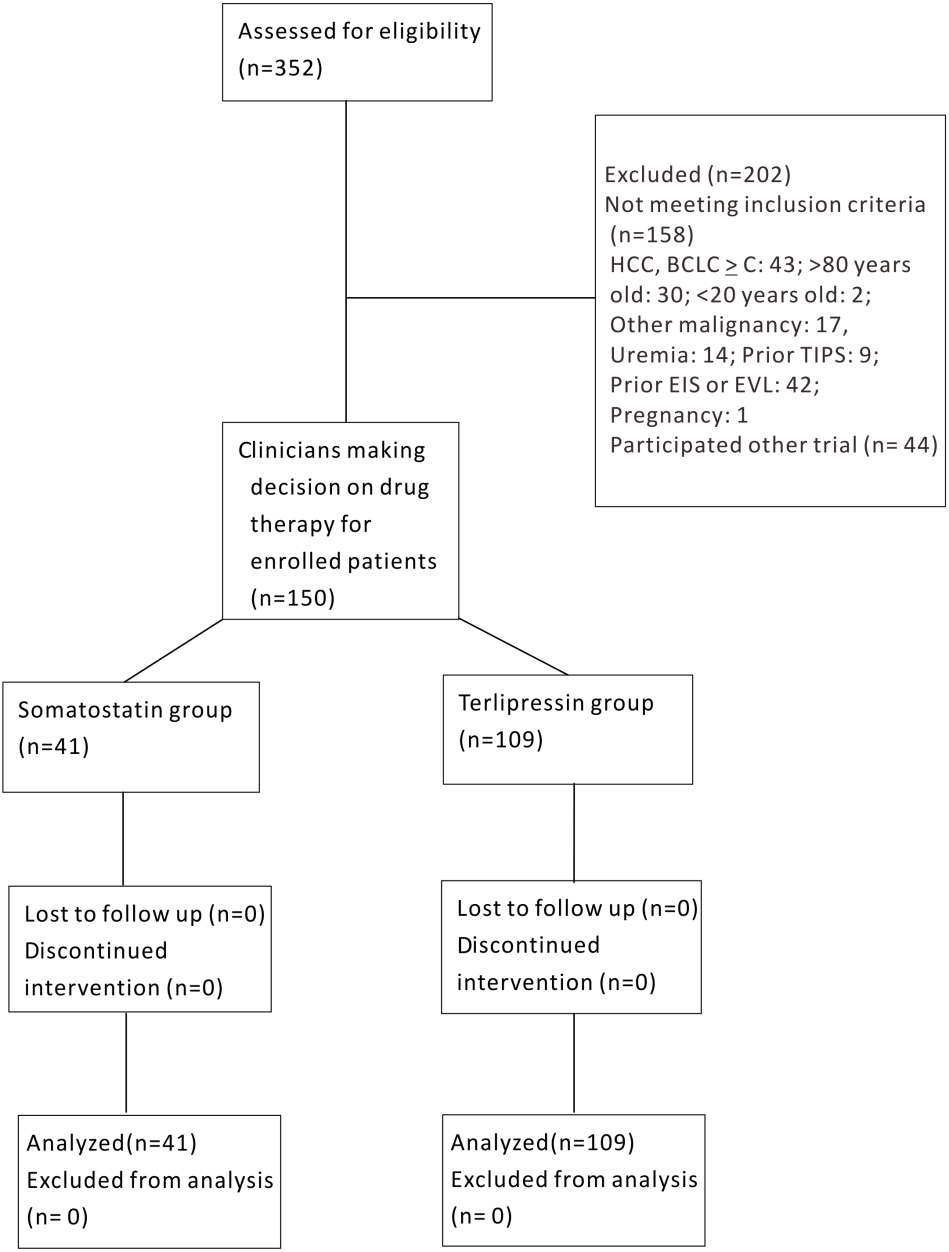
Schematic flow chart of enrollment for somatostatin and terlipressin groups in acute esophageal variceal bleeding patients.

Of the 352 patients screened, 202 patients were excluded owing to HCC BCLC ≥ C (*n* = 43), >80 or <20 years old (*n* = 32), other malignancies (*n* = 17), uremia (*n* = 14), prior TIPS (*n* = 9), prior EIS or EVL (*n* = 42), pregnancy (*n* = 1) and refusal to participate (*n* = 44). Finally, a total of 150 patients were enrolled in the trial, with 41 in group S and 109 in group T (Fig. 1).

The baseline demographic characteristics including gender, age, etiology of cirrhosis, symptoms, Child Pugh’s scores and MELD scores were comparable in both treatment groups, all *P* > 0.05 (Table 1). Likewise, the endoscopic findings were not different in terms of variceal size, red color signs and presence of gastric varix and portal hypertensive gastropathy between the two study groups (Table 1).

**Figure 2:**
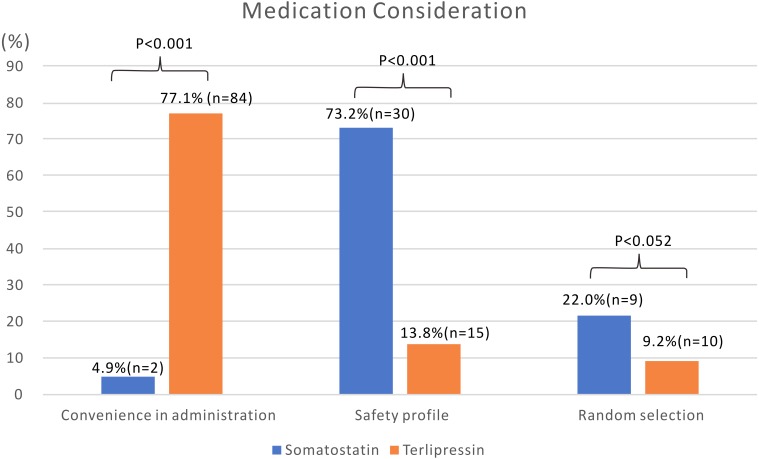
Medication consideration of physicians when prescribing somatostatin or terlipressin for patients with acute esophageal variceal bleeding.

Convenient administration (77.1%, *n* = 84) followed by a good safety profile (13.8%, *n* = 15) were the main reasons for physician drug choice in group T (Fig. 2). On the other hand, good safety profile (73.2%, *n* = 30) was a major consideration for physicians in group S. Inconvenient administration (4.9%, *n* = 2) was a major limitation for physician choice in group S.

Main clinical treatment results and adverse events are shown in Table 2. No participants were lost during follow up. Only one patient in each study group was considered as treatment failure as per definition. Very early rebleeding within 49–120 h occurred in one patient in each group (*p* = 0.469). Four patients (10.0%) in group S and 14 patients (13.0%) in group T had EV rebleeding episodes within 6–42 d (*p* = 0.781). No significant differences were observed in 6-week mortality rate (9.8% vs. 12.8%, *p* = 0.419), blood transfusion (4.6 ± 5.7 vs. 4.0 ± 3.6 units, *p* = 0.421) and hospital stay (8.1 ± 2.8 vs. 8.9 ± 4.0 days, *p* = 0.240).

**Table 1:** Baseline demographic characteristics of somatostatin and terlipressin groups at entry of current study.

	Somatostatin group (*N* = 41)	Terlipressin group (*N* = 109)	*P* value
Men/Women	32/9	77/32	0.416
Age (years)	55	57	0.351
Etiology of cirrhosis			0.438
Alcoholism	18 (43.9%)	34 (31.2%)	
Hepatitis B	11 (26.8%)	30 (27.5%)	
Hepatitis C	7 (17.1%)	30 (27.5%)	
Hepatitis B + Hepatitis C	5 (12.2%)	15 (13.8%)	
Symptoms (hematemesis/melena/both)	20∕10∕11	44∕24∕39	0.578
Albumin (g/dL)	3.1 ± 0.5	3.1 ± 0.6	0.446
Bilirubin (mg/dL)	2.5 ± 2.8	2.8 ± 5.0	0.486
Sodium (mmol/L)	136.7 ± 5.6	137.8 ± 5.1	0.287
Creatinine (mg/dL)	1.3 ± 1.5	1.1 ± 0.7	0.476
Ascites present	25 (61.0%)	53 (48.6%)	0.202
Prothrombin time (s)	3.1 ± 1.9	2.9 ± 2.9	0.752
Encephalopathy	8 (19.5%)	22 (20.2%)	0.563
Child-Pugh score	7.9 ± 1.6	7.6 ± 1.9	0.389
Child-Pugh class	9∕23∕7	34∕56∕17	0.622
MELD score	12.0 ± 5.2	11.0 ± 4.9	0.270
Coexisting HCC	13 (31.7%)	39 (35.8%)	0.703
Variceal size			0.605
F1	2 (4.9%)	7 (6.4%)	
F2	21 (51.2%)	64 (58.7%)	
F3	18 (43.9%)	38 (34.9%)	
Red color signs			0.672
Mild	15 (36.6%)	46 (42.2%)	
Moderate	19 (46.3%)	50 (45.9%)	
Severe	7 (17.1%)	13 (11.9%)	
Presence of gastric varix	10 (24.4%)	33 (30.3%)	0.310
Presence of PHG	11 (27.5%)	22 (20.2%)	0.230

**Notes.**

HCC hepatocellular carcinoma MELD model for end stage liver disease PHG portal hypertensive gastropathy

**Table 2:** Main clinical treatment results and adverse events in somatostatin and terlipressin groups.

	Somatostatin group (*N* = 41)	Terlipressin group (*N* = 109)	*P* value
Initial hemostasis (within 48 h)	40 (97.6%)	108 (99.1%)	0.473
Rebleeding			
Very early rebleeding (49–120 h)	1 (2.5%)	1 (0.9%)	0.469
Early rebleeding (6–42 d)	4 (10.0%)	14 (13.0%)	0.781
–Sources of early rebleeding			
Esophageal varices	3 (7.5%)	10 (9.3%)	1.000
EVL-induced ulcer	1 (2.5%)	4 (3.7%)	1.000
Gastric varices/PHG	0 (0.0%)	0 (0.0%)	1.000
Mortality at 6 wk	4 (9.8%)	14 (12.8%)	0.419
–Cause of death			
EV bleeding	3 (2.8%)	7 (6.4%)	1.000
Liver failure	0 (0%)	3 (2.8%)	0.562
Bacterial peritonitis	1 (2.4%)	2 (1.8%)	1.000
Hepatorenal syndrome	0 (0%)	1 (0.9%)	1.000
Pneumonia	0 (0%)	1 (0.9%)	1.000
Blood transfusion (unit)	4.6 ± 5.7	4.0 ± 3.6	0.421
Hospital stay (day)	8.1 ± 2.8	8.9 ± 4.0	0.240
Probable adverse events	13 (31.7%)	36 (33.0%)	0.878
–Vasoactive agent-related			
Chest tightness/pain	6 (14.6%)	23 (21.1%)	0.488
Hypertension (SBP >140 mmHg)	5 (12.2%)	26 (23.9%)	0.116
Abdominal pain	2 (4.9%)	12 (11.0%)	0.352
Arrhythmia	1 (2.4%)	4 (3.7%)	1.000
Hyponatremia (Na <125 mmol/L)	2 (4.9%)	18 (16.5%)	0.103
Lymphangitis	0 (0%)	6 (5.5%)	0.189
Renal failure (Cr >2 mg/dL)	4 (9.8%)	3 (2.8%)	0.089
Hyperglycemia (Glu >300 mg/dL)	2 (4.9%)	4 (3.7%)	0.665
Flush	1 (2.4%)	3 (2.8%)	1.000
–EVL-associated			
Chest tightness/pain	6 (14.6%)	23 (21.1%)	0.488
Retrosternal pain	5 (12.2%)	18 (16.5%)	0.617
Dysphagia/odynophagia	1 (2.4%)	2 (1.8%)	1.000
Post-EVL ulcer	4 (9.8%)	13 (11.9%)	1.000
Esophageal stricture	0 (0%)	0 (0%)	1.000

**Notes.**

EV esophageal varix EVL endoscopic variceal ligation PHG portal hypertensive gastropathy SBP systolic blood pressure

Overall adverse events were classified into two subgroups, either vasoactive agent-related or EVL-associated (Table 2). In the former subgroup, chest tightness/pain (14.6% vs. 21.1%), hypertension (12.2% vs. 23.9%), abdominal pain (4.9 vs. 11.0%), arrhythmia (2.4% vs. 3.7%), lymphangitis (0% vs. 5.5%), hyperglycemia (4.9% vs. 3.7%) and flushing (2.4% vs. 2.8%) were comparable in both groups S and T. Patients in group S vs. group T had shown a trend of having more renal failure (group S: 9.8% vs. group T: 2.8%, *p* = 0.089) but group T vs. group S had shown a trend towards more hyponatremia (group S: 4.9% vs. group T: 16.5%, *p* = 0.103). In the later subgroup, all the EVL-associated symptoms, such as chest tightness/pain (group S: 14.6% vs. group T: 21.1%), retrosternal pain (12.2% vs. 16.5%), dysphagia/odynophagia (2.4% vs. 1.8%), post-EVL ulcer (9.8% vs. 11.9%) and esophageal stricture (0% vs. 0%) were similar between groups S and T (all *P* values >0.05).

## Discussion

Rebleeding is a major determinant of poor outcome following initial control of acute EVB. Nowadays, combined pharmacological and endoscopic therapy is key to management of acute EVB in real-world clinical practice. Vasoactive drug therapy should be given as soon as possible and ideally before endoscopy. A few studies have been conducted thus far that compares the efficacy of terlipressin vs. somatostatin vs. octreotide in acute EBV with a short infusion duration of 3 days up to 5 days (Seo et al., 2014; Abid et al., 2009). The current study shows that a 3-day terlipressin treatment was as effective as a 3-day somatostatin treatment in controlling initial bleeding but also preventing early rebleeding. Besides, we have demonstrated that most clinicians would choose terlipressin over somatostatin because of convenience. However, safety profile was a major consideration of physicians who preferred somatostatin.

Most guidelines, including the Baveno IV guidelines, have suggested the use of vasoactive agents for 2–5 days (De Franchis, 2005; Farooqi et al., 2007). The most recent Baveno VI guidelines recommended a 5-day use of vasoactive agents (De Franchis, 2015). However, the optimal duration for the use of vasoactive agents in acute EVB remains inconclusive thus far. Previous clinical trials have shown that three days of somatostatin and two days of octreotide were not inferior to five days of both drugs in preventing early rebleeding (Rengasamy et al., 2015; Li et al., 2015). On the other hand, early administration of vasoactive agents especially the first two days of acute EVB is probably preferred. This is because of a greater increase of portal pressure after acute EVB as observed by Avgerinos A et al. with subsequent return to pretreatment level after the first two days after EVB (Avgerinos et al., 2004). In Taiwan, all patients with acute EVB would be given a 3-day course of vasoactive agents because this is the maximal duration that is fully covered by the NHI system. However, the current study showed that despite giving a shorter 3-day course it did not affect initial hemostatic control and also early rebleeding. Our findings support the Avgerinos observation that control of portal pressure for the first two days is most important.

The most recent meta-analysis did not show a difference with either vasopressin/terlipressin or somatostatin/octreotide in the prevention of very early rebleeding and early rebleeding (Wang et al., 2015). The 5-day variceal rebleeding rates of our current study were 0.9% in group T and 2.5% in group S, and these rates were similarly to previous studies (Seo et al., 2014; Wang et al., 2015). However, very early rebleeding rate was lower in our study if compared to Seo et al. (2014) despite a shorter duration (3-day vs. 5-day) of therapy. Two probable reasons may explain this difference. First, the recruitment of patients with gastric variceal bleeding may result in a higher very early rebleeding rate because the treatment success of gastroesophageal varices type 2 and isolated gastric varices was known to be lower compared to esophageal varices and gastroesophageal varices type 1, i.e., 55–87% versus 88–91% respectively (Seo et al., 2014). Secondly, the treatment effect of EVL is far more prominent than vasoactive agents if both are used in combination (Lo et al., 2009; Kumar et al., 2015).

Terlipressin is currently the only drug that has been shown to have survival benefit with a 34% relative risk reduction in mortality, unlike somatostatin (Lo, 2010; Ioannou, Doust & Rockey, 2003; Abraldes & Bosch, 2002). It is for this reason that terlipressin is considered as the first choice based on a number of expert opinions (Abraldes & Bosch, 2002; Dell’Era, De Franchis & Iannuzzi, 2008). Besides that, a randomized trial from the South Korea had reported that the effect of terlipressin in reducing portal hypertension was more sustained compared to octreotide (Baik et al., 2005). Furthermore, when compared with somatostatin, terlipressin has been shown to improve thoracic blood volume, liver reserve blood volume and also improve the hyperdynamic state in cirrhosis (Kalambokis et al., 2010). These effects are particularly beneficial in patients with advanced liver diseases and acute EVB episode. Indirect evidence suggested that terlipressin might prevent hemorrhage-induced renal impairment, and its liver protection effect has been reported in cases of septic shock in an animal model (Moreau et al., 2002).

A global study found that 55% of physicians preferred terlipressin compared to 38% who favored octreotide (*p* < 0.001)  (Rahimi, Guntipalli & Rockey, 2014). Approximately two-thirds of the patients were prescribed with terlipressin in our current study. Noticeably, the current unavailability of terlipressin is the issue of why most physicians worldwide do not prescribe it, and overall 93% of those physicians would have like to use it if it is readily offered, particularly in the North America  (Rahimi, Guntipalli & Rockey, 2014). The actual reason why physicians were more willing to use terlipresin in the global study is unclear and our study aimed to address this.

Convenience of administration was the main consideration by our clinicians who chose terlipressin in the current study. Terlipressin infusion using slow intravenous push every 6 hourly is relatively easier and is more favored by nurses if compared to continuous infusion with somatostatin. Conversely, side effects of terlipressin may be an issue in daily clinical practice of physicians (approximately one third had side effects in the current study). Terlipressin two mg every four hours in the first 1–2 days followed by one mg for the following 2–5 day is the current recommendation, whereas the two mg bolus injection followed by one mg infusion every six hours in our current protocol resulted in a lower total dosage given but with comparable efficacy and less side effects (Augustin, Gonzalez & Genesca, 2010).

The safety profile is a top priority of clinicians who chose somatostatin. For patients with ischemic heart diseases or uncontrolled hypertension, somatostatin may be a good alternative to terlipressin. Somatostatin has the advantage of having lesser side-effects including hypertension and hyponatremia. A previous study also showed that compared to terlipressin, somatostatin had fewer side effects (21% vs. 29%, not statistically significant (D’Amico, Pagliaro & Bosch, 1999).

Comparing the costs of drugs for the duration of three days, the total cost of terlipressin and somatostatin were USD 621.32 and USD 496.43 respectively. An additional USD 124.89 or 20% of expenditures are needed for terlipressin. The cost factor may be neglected by clinicians in real-world medical practice because expenditure of both drugs is fully subsidized by the health care plan in Taiwan. Therefore, convenience of use outweighs the cost factor in real world in Taiwan.

There were limitations in our study. Firstly, selection bias might exist in both treatment groups due to clinicians’ preference of one drug over the other although this reflects the real-world clinical practice. Secondly, although a single institution study might not reflect the practice of other institutions in Taiwan but the similar NHI program implemented country-wide provided some consistencies in management across different institutions. Lastly, referral bias might occur in any tertiary referral centers where only patients with a greater tendency to stop bleeding spontaneously were able to arrive at the tertiary hospital.

## Conclusion

In real-world clinical practice, most of the physicians preferred terlipressin over somatostatin because of convenience in administration although terlipressin is more expensive and its safety remains a concern. Yet, the efficacy on hemostasis and safety does not seem to differ significantly between patients with acute EVB who received short three-day course of either somatostatin or terlipressin as an adjuvant to EVL.

##  Supplemental Information

10.7717/peerj.7913/supp-1 Supplemental Information 1 Data in SPSS format

## Abbreviations

EVL: endoscopic variceal ligation
EVB: esophageal variceal bleeding
NHI: National Health Insurance
HCC: hepatocellular carcinoma
BCLC: Barcelona-Clinic Liver Cancer
COPD: chronic obstructive pulmonary disease
EIS: endoscopic injection sclerotherapy
TIPS: transjugular intrahepatic porto-systemic stent shunt
group T: terlipressin and group
S: somatostatin

## Additional Information and Declarations

### Data Availability

The following information was supplied regarding data availability: Raw data is available as a Supplemental File.
